# Improvement in the Water Retention Characteristics of Sandy Loam Soil Using a Newly Synthesized Poly(acrylamide-*co*-acrylic Acid)/AlZnFe_2_O_4_ Superabsorbent Hydrogel Nanocomposite Material

**DOI:** 10.3390/molecules17089397

**Published:** 2012-08-03

**Authors:** Shaukat Ali Shahid, Ansar Ahmad Qidwai, Farooq Anwar, Inam Ullah, Umer Rashid

**Affiliations:** 1 Department of Physics, University of Agriculture Faisalabad 38040, Pakistan; 2 Department of Physics, University of Karachi, Karachi 75270, Pakistan; 3 Department of Chemistry, University of Sargodha, Sargodha 40100, Pakistan; 4 Department of Chemistry and Biochemistry, University of Agriculture Faisalabad 38040, Pakistan; 5 Institute of Advanced Technology, Universiti Putra Malaysia, Serdang 43400, Selangor, Malaysia

**Keywords:** poly(AAm-*co*-AA)/AlZnFe_2_O_4_ superabsorbent hydrogel nanocomposite characterization, EDX, FESEM, FTIR, soil amendment, seedling growth

## Abstract

The use of some novel and efficient crop nutrient-based superabsorbent hydrogel nanocomposites (SHNCs), is currently becoming increasingly important to improve the crop yield and productivity, due to their water retention properties. In the present study a poly(Acrylamide-*co*-acrylic acid)/AlZnFe_2_O_4_ superabsorbent hydrogel nanocomposite was synthesized and its physical properties characterized using Energy Dispersive X-ray (EDX), FE-SEM and FTIR spectroscopic techniques. The effects of different levels of SHNC were studied to evaluate the moisture retention properties of sandy loam soil (sand 59%, silt 21%, clay 19%, pH 7.4, EC 1.92 dS/m). The soil amendment with 0.1, 0.2, 0.3 and 0.4 w/w% of SHNC enhanced the moisture retention significantly at field capacity compared to the untreated soil. Besides, in a separate experiment, seed germination and seedling growth of wheat was found to be notably improved with the application of SHNC. A delay in wilting of seedlings by 5–8 days was observed for SHNC-amended soil, thereby improving wheat plant growth and establishment.

## 1. Introduction

The shortage of irrigation water, drought, deforestation and soil erosion are one of the major problems of agriculture around the globe. Prolonged periods of drought can cause environmental, agricultural and economic problems leading to social unrest and humanitarian crisis. The development of non-traditional new technologies to conserve water is becoming important for attaining a sustainable economic growth, especially in agricultural countries. 

In recent years, crosslinked polymers with hydrophilic nature, known as superabsorbent polymers (SAPs), have attracted much interest in this area as they can improve water and fertilizer retention in soil and thus increase plant growth [1]. Moreover, soil amendments with SAPs can improve plant establishment in drought prone areas [2]. Superabsorbent polymers (SAPs) can absorb significant amounts (up to 2,000 g/g) of water, therefore are considered very suitable for applications in horticulture and agriculture [3,4,5,6,7,8]. The desired features of superabsorbents for agriculture purposes include high swelling rate, swelling capacity, and reswellibility. These features are dependent on structure of the polymer network, particle size and porosity. The larger surface area of swollen particles can provide more voids in the soil matrix which can enhance soil breathing [3,4]. 

There are several approaches to prepare hydrogel nano-composite structures. Several clays such as kaolin [9], bentonite [10], montmorillonite [11], potassium humate [12], sodium humate [13,14], attapulgite [15], smectite [16] and cellulose nanowhiskers [17,18] have so far been used in the synthesis of superabsorbent composites (SHCs). Use of humic compounds in soil improves absorption of microelements, photosynthesis, root development and soil structure [14]. Currently most of the research in the area of development of polymers for soil water conservation involves the use of natural clays as nanocomposite materials to enhance only the physical properties of the superabsorbent hydrogels without considering the actual crop nutrient requirements. Extensive studies with cereals by Cakmak [19] in Turkey showed that the problem of Zn deficiency was widespread, with serious implications, not only for crop production, but also for human health. Zinc (Zn) and Iron (Fe) are essential micronutrients which enhance the yield as well as quality of the crops. The protein contents of wheat are significantly improved with Zn and Fe application [19,20]. 

A larger surface area of nutrients offers greater chance for the plant roots to encounter Zn fertilizer [21]. Hence, there is much need to explore some specific and efficient crop nutrient-based superabsorbent hydrogel nanocomposites to not only enhance the soil water retention capacity and porosity, but also to simultaneously improve the crop yield and productivity. In the present study the effects of different levels of a newly synthesized poly(acrylamide-*co*-acrylic acid)/AlZnFe_2_O_4_ superabsorbent hydrogel nanocomposite (SHNC) on the moisture retention characteristics of sandy loam soils as well as on the growth of wheat (*Triticum aestivum* L.) in SHNC amended soil were appraised. 

## 2. Results and Discussion

### 2.1. Characterization of SHNC

**Figure 1:**
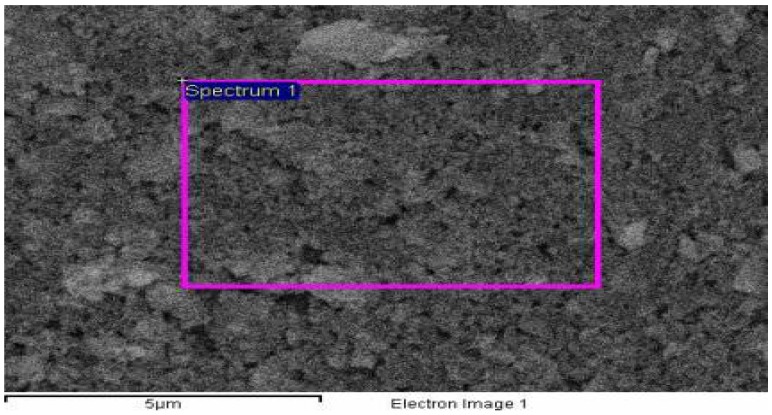
FE-SEM analysis of the AlZnFe_2_O_4_.

**Figure 2:**
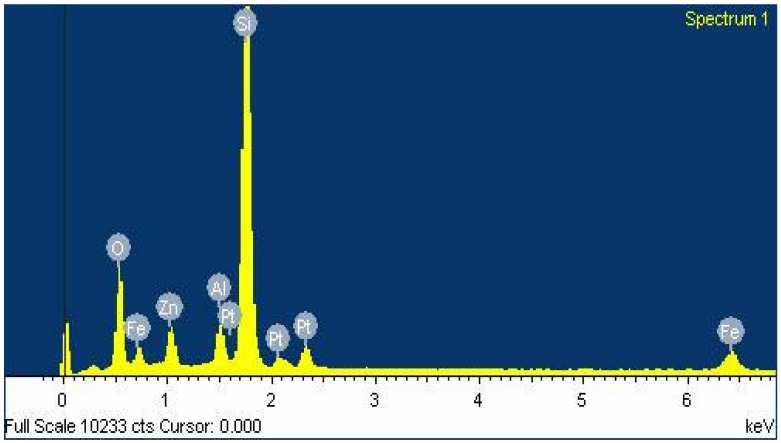
EDX spectrum of AlZnFe_2_O_4_ nanocomposite.

**Figure 3:**
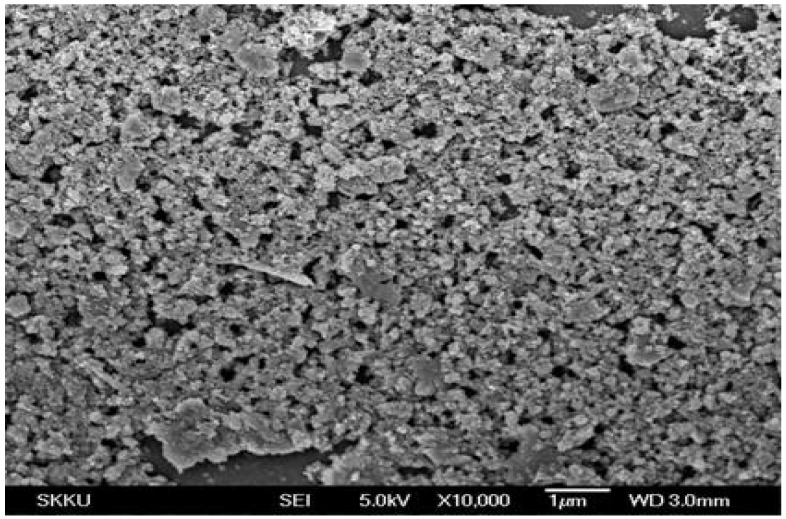
Scanning electron micrograph of AlZnFe_2_O_4_ nanocomposite.

Keeping in view the soil moisture retention and micronutrient requirements, a novel poly(acrylamide-*co*-acrylic acid)/AlZnFe_2_O_4_ superabsorbent hydrogel nanocomposite was synthesized for agricultural use. FE-SEM analysis of the as-synthesized nanoparticles was carried out and the results are shown in Figure 1. The FE-SEM analysis showed that nanoparticles have a mean diameter of 50 nm. The EDX spectrum of the nanoparticles shows that nanoparticles contain only Fe, Zn, Al and O with no trace of by-products (Figure 2). The Scanning Electron Micrograph of AlZnFe_2_O_4_ nanocomposite also shows the particle size to be around 50 nm (Figure 3). 

#### 2.1.1. FTIR Spectroscopy

**Figure 4:**
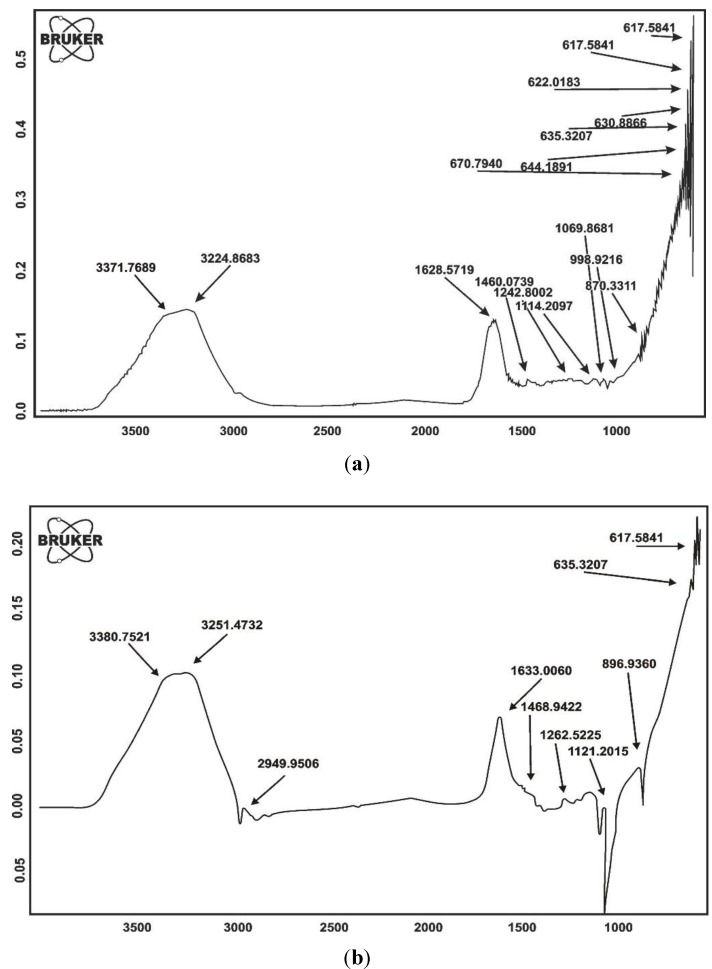
(**a**) FTIR spectrum of poly(acrylic acid-*co*-acrylamide); (**b**) FTIR spectrum of poly(Acrylamide-*co*-acrylic acid)/AlZnFe_2_O_4_.

It is evident from the FTIR spectrum of the superabsorbent hydrogel that two N–H stretching bands appear at 3224.8683 and 3371.7689 cm^−1^, respectively. The stretching of C=O is also observed at 1682.1121 cm^−1^. The peak at 1460.0739 cm^−1^ is the stretching band of C–N and 1114.2097 cm^−1^ is another peak related to amide group. The peak appearing at 1242.8002 cm^−1^ is the characteristics (C–O) stretching peak of –COOH (Figure 4a).

When the spectrum of SHNC is compared with that of superabsorbent hydrogel, it can be observed that the N–H stretching band of the –NH_2_ group at 3224.8663 and 3371.7689 cm^−1^ is shifted to 3251.4732 and 3380.7521 cm^−1^, respectively while the stretching of C=O at 1628.5719 cm^−1^, C–N at 1460.0739 and C–O at 1242.8002 cm^−1^ is shifted to 1633.0060, 1468.9422 and 1262.5225 cm^−1^, respectively indicating relatively weaker intensity of peaks due to composite formation (Figure 4b).

### 2.2. Water Absorption by SHNC

**Figure 5:**
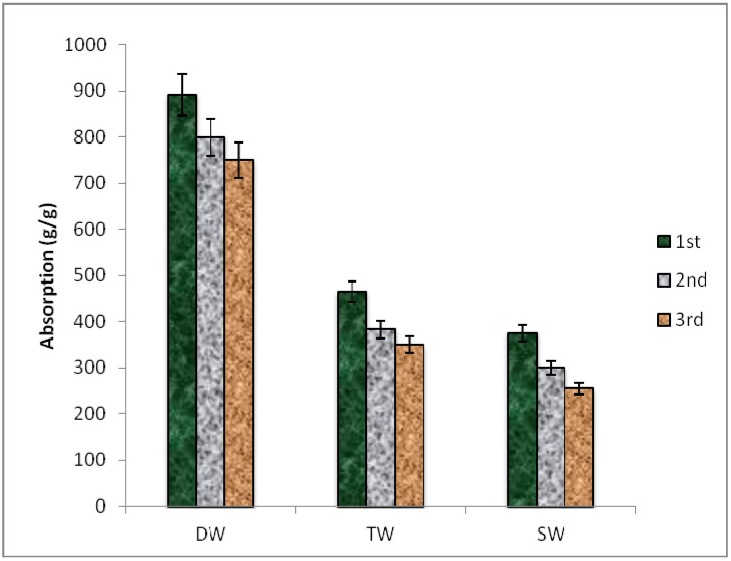
Absorption of distilled water (DW), tap water (TW) and saline water (SW) by SHNC during 1st, 2nd and 3rd wetting and drying cycles.

The maximum quantity of water absorbed by SHNC was 892 g/g for distilled water, 471 g/g for tap water and 390 g/g for saline water during its 1st hydration cycle (Figure 5). The absorption of water by SHNC was thus found to be faster in distilled water as compared to tap and saline water, and the maximum absorption was achieved in 5, 7 and 14 h. in distilled, tap and saline water, respectively. Similar trends of water uptake by hydrogels synthesized from a mixture of *N,N*-methylene-bis-acrylamide, Na and K salts of acrylic acid was observed by Akhtar *et al.*, [2], however, the maximum water uptake was 505 g/g in distilled water followed by 212 g/g by tap water and 140 g/g by saline water during the first hydration cycle. The decrease in water absorbency during the 2nd and 3rd hydration cycle can be attributed to the fact that Ca^+2^, Mg^+2^ and other ions (present in water) form insoluble salts with acrylic acid. These ions attach permanently to the carboxylic acid groups of acrylic acid blocking the active negative sites of the hydrogel and decreasing the absorbency of the hydrogel. As the polymer in the present research is a copolymer of acrylic acid and acrylamide (95% and 5%, respectively), for technical understanding it could be described that the acrylic acid moieties present in the polymeric chain are more hydrophilic than that those of the other co-monomer. The ionization of acrylic acid as acrylate ions further increases its affinity for water absorption. Once the polymers is fully absorbed the, polyvalent cations such as Ca^+2^ and Mg^+2^ present in water produce relatively stable acrylic acid carboxylates. The salts have poor ionization power thus reducing the water absorbing capacity of the polymer on rehydration after one soaking and drying cycle. This is also supported by the consistency of the behavior in distilled water. However, the enhanced water holding capacity of SHNC might be linked to the presence of AlZnFe_2_O_4_ nanocomposite in the superabsorbent hydrogel polymeric network and can be attributed to the fact that the cations on the surface are ionized and dispersed in the superabsorbent hydrogel and enhance the hydrophilicity of SHNC. In agreement with the present investigation, Spagnol *et al.* [17,18,22], in their studies on hydrogels filled with cellulose nanowhiskers, also observed improvement in swelling capacity. Hydrogel immersion in sodium and calcium salt solutions while investigating the reswelling capacity of SHNC during 2nd and 3rd hydration cycle is also reported in the literature [18]. 

### 2.3. Effect of SHNC on Moisture Retention in Soil

**Figure 6:**
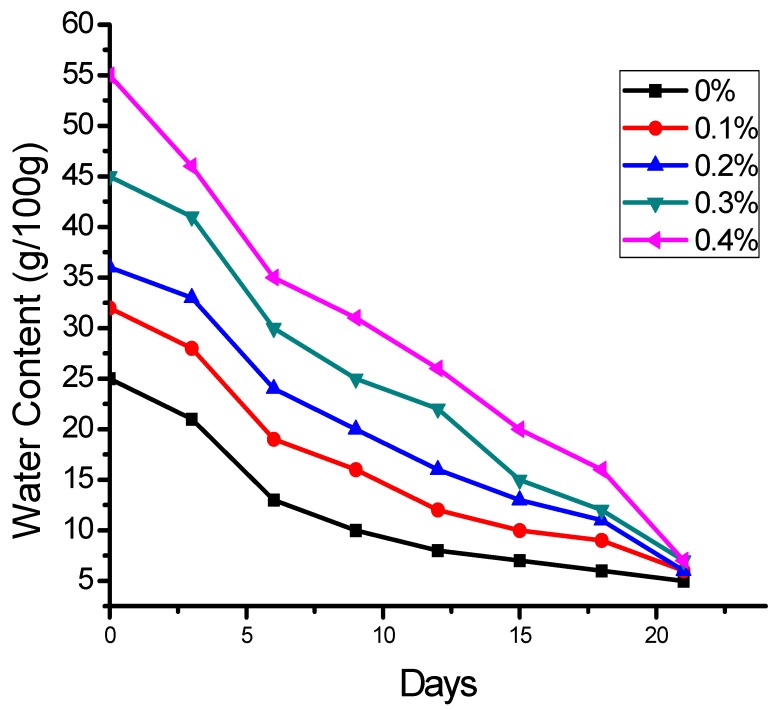
Water retention by the soil amended with different levels (0.1, 0.2, 0.3, 0.4%) of SHNC at field capacity.

The water retention by the amended soil at field capacity (0.03 MPa pressure) is shown in Figure 6 which indicates that the water retention of the soil depends on the quantity of the SHNC used and was increased up to approximately 60 to 100% at field capacity with the application of 0.1 to 0.4 w/w% of the newly synthesized poly (Acrylamide-*co*-acrylic acid)/AlZnFe_2_O_4_ superabsorbent hydrogel nanocomposite. Similar effects on soil water holding properties of loamy soil were reported by Akhtar *et al.* [2], in sandy loam and loam soil. Bai *et al.*, [23] studied the effects of super-absorbent polymers on water retention and other physical and chemical properties of soil following different wetting and drying cycles and observed a decrease in efficiency during the second and third wetting and drying cycles. 

The water retention in the soil was increased with the increase in the concentration of SHNC and highest value of moisture retention was achieved with the addition of 0.4 w/w% SHNC to the soil. In comparison to our present value, Dorraji *et al.* [24] achieved moisture retention as high as 0.6 w/w% with the use of hydrophilic polymer in sandy and loamy soils.

### 2.4. Effect of SHNC on Soil pH and Electrical Conductivity (EC)

Soil pH and EC are important factors in the chemical, physical and biological properties of soils [23]. The pH and EC varied with the application of SHNC. Soil pH was reduced by 1.49, 2.43, 3.78 and 4.86% at concentration of 0.1, 0.2, 0.3 and 0.4%, respectively, compared with the control. Meanwhile, the electrical conductivity (EC) was increased with SHNC application. The EC of SHNC-amended soil increased about 6, 11, 45 and 56% as compared to the control at concentrations of 0.1, 0.2, 0.3 and 0.4 w/w%. Bai *et al.*, [23], while studying the characteristics of chitosan-graft-poly(acrylic acid)/sodium humate superabsorbent, observed a similar effect on the pH and EC of soils as noted in the recent investigation and linked this phenomenon to the chemical structure of the superabsorbent polymer and characteristics of the soils. 

### 2.5. Effect on Soil Bulk Density and Porosity

The soil bulk density varied with soil moisture (Table 1) and decreased by 3.42, 4.10, 7.53 and 13.57% at SHNC concentrations of 0.1%, 0.2%, 0.3% and 0.4%, respectively. In contrast soil porosity increased by 5.71, 11.43, 22.86 and 31.43% at SHNC concentrations of 0.1%, 0.2%, 0.3% and 0.4%, respectively. (Table 1) Previously, an increase in the average dimension of porous with the addition of nanofibrils into the hydrogel matrix was observed by Spagnol *et al*. [22].

**Table 1:** Effect of different levels of SHNC on physical properties of sandy loam soil.

Hydration	SHNC conc.(%)	Soil pH	Electrical Conductivity (EC) dS/m	Porosity %	Hydraulic Conductivity mm/h	Bulk Density g/cm^3^
	0	7.40 ± 0.14	1.73 ± 0.21	33.50 ± 1.73	74.92 ± 2.50	1.46 ± 0.10
	0.1	7.31 ± 0.27	1.83 ± 0.15	35.35 ± 1.92	62.40 ± 2.24	1.41 ± 0.05
1st	0.2	7.22 ± 0.12	1.92 ± 0.23	37.17 ± 2.12	47.32 ± 1.65	1.40 ± 0.14
	0.3	7.12 ± 0.14	2.18 ± 0.16	41.60 ± 1.82	38.25 ± 1.64	1.35 ± 0.06
	0.4	7.04 ± 0.21	2.73 ± 0.35	44.21 ± 2.30	27.33 ± 1.28	1.27 ± 0.05
	0	7.40 ± 0.12	1.73 ± 0.11	33.30 ± 1.13	74.92 ± 2.40	1.46 ± 0.15
	0.1	7.35 ± 0.21	1.83 ± 0.12	35.14 ± 1.72	61.40 ± 2.23	1.42 ± 0.09
2nd	0.2	7.29 ± 0.11	1.91 ± 0.20	37.12 ± 2.12	44.32 ± 1.55	1.40 ± 0.12
	0.3	7.22 ± 0.10	2.15 ± 0.16	40.50 ± 1.22	40.25 ± 1.34	1.38 ± 0.07
	0.4	7.17 ± 0.19	2.47 ± 0.32	42.21 ± 2.35	37.33 ± 1.18	1.32 ± 0.12
	0	7.40 ± 0.11	1.73 ± 0.21	33.54 ± 1.23	74.92 ± 2.30	1.46 ± 0.11
	0.1	7.36 ± 0.20	1.77 ± 0.19	34.45 ± 1.22	69.40 ± 2.14	1.45 ± 0.10
3rd	0.2	7.32 ± 0.11	1.82 ± 0.21	36.37 ± 2.11	59.32 ± 1.45	1.42 ± 0.11
	0.3	7.31 ± 0.12	2.08 ± 0.12	38.20 ± 1.42	49.25 ± 1.34	1.39 ± 0.26
	0.4	7.24 ± 0.20	2.33 ± 0.34	40.11 ± 2.20	40.33 ± 1.21	1.37 ± 0.19

The results are means of three replicates.

### 2.6. Effect of SHNC on Hydraulic Conductivity

Another important indicator of soil quality is the hydraulic conductivity of the soil which was initially observed to be 74 mm/h (Table 1). The addition of 0.1, 0.2, 0.3 and 0.4% of SHNC reduced the hydraulic conductivity by 16%, 36%, 48% and 63%, respectively. The decrease in hydraulic conductivity was in agreement with a former study by El-Shafei *et al.* [25].

### 2.7. Effect of SHNC on Seed Germination and Seedling Growth of Wheat

Soil water potential strongly affects wheat seedling emergence and drought could be harmful to seedling establishment [25]. The enhancement in soil moisture retention and improvement in seed germination and seedling growth as a result of superabsorbent hydrogel amendments have been reported in the literature, however with varying superabsorbent hydrogel materials [2]. In the present study the seed germination was considerably higher in 0.3 and 0.4% SHNC amended soils as compared to control, however the other levels of SHNC did not exhibit notable effects. Meanwhile, wheat seedling growth was enhanced by the addition of SHNC. Besides, shoot length of wheat was significantly higher at 0.3 and 0.4% SHNC compared with other SHNC levels or control. Shoot length of wheat increased considerably with the addition of SHNC. The addition of 0.3% and 0.4% SHNC in sandy loam soil also significantly increased the fresh and dry weights of wheat shoots (Table 2). 

**Table 2:** Effect of different levels of SHNC on seed germination and seedling growth (21 days) of wheat (*Triticum aestivum* L.) in sandy loam soils.

SHNC Level (%)	Seed Germination (%)	Shoot Length (cm)	Shoot Fresh Weight (mg)	Shoot Dry Weight (mg)
0	80.2 ± 1.73 ^a^	16.24 ± 1.91 ^b^	88.75 ± 2.45 ^c^	18.11 ± 1.91 ^b^
0.1	91.5 ±1.82 ^ab^	20.76 ± 1.82 ^ab^	108.55 ± 3.25 ^b^	20.45 ± 1.82 ^b^
0.2	92.0 ± 1.90 ^a^	22.61 ± 2.05 ^ab^	118.35 ± 3.62 ^a^	24.50 ± 1.91 ^a^
0.3	92.4 ± 2.12 ^ab^	24.36 ± 2.15 ^a^	133.72 ± 4.50 ^a^	27.20 ± 2.10 ^a^
0.4	96.1 ± 2.30 ^ab^	25.80 ± 2.23 ^a^	140.00 ±5.24 ^ab^	32.42 ±2.31^ab^

Different superscript letters indicate significant difference at *p* = 0.05. Mean with same alphabets with in the same column indicate non significant (*p* > 0.05) differences among SHNC concentrations.

### 2.8. Effect of SHNC on Permanent Wilting Point

The maintenance of adequate soil moisture is a necessary pre-requirement for soil water retention for agriculture purposes. The soil amendment with SHNC increased the moisture content at field capacity of sandy loam soils. At the same time, the amendment with SHNC decreased the hydraulic conductivity and slowed the rate of soil moisture loss that delayed wilting of seedlings grown. The onset of permanent wilting point (PWP) was delayed by 5, 6, 7 and 8 days with SHNC concentrations of 0.1, 0.2, 0.3 and 0.4 w/w%, respectively. A similar effect on the wilting point has been reported by Akhtar *et al.* [2].

### 2.9. General Discussion

In the present study the preliminarily water absorption capacity of the newly synthesized superabsorbent hydrogel nanocomposite (SHNC) was investigated using distilled, tap and saline water for three hydration cycles. In the first hydration cycle, SHNC exhibited remarkable water holding capacity which decreased slightly during the second and third cycles (Figure 5). This decrease in second and third hydration cycle was previously recorded by Akhtar *et al.*, [2] during a study with a simple hydrogel. However, the water absorbency of newly synthesized SHNC was much more (892 g/g) than previously observed [2,6,8,25,26,27,28,29]. This appreciable water holding capacity might be linked to the introduction of a very small amount of AlZnFe_2_O_4_ nanocomposite in the superabsorbent hydrogel polymeric network and can be attributed to the fact that the cations on the surface ionized and dispersed in the superabsorbent hydrogel and thus enhanced the hydrophilicity of SHNC. The previous studies reported in the literature verify this phenomenon [2,23,27,28,29] and illustrate that water absorbency of a superabsorbent hydrogel depends on cross linking density, affinity of the superabsorbent hydrogel to water and ionic osmotic pressure [22]. 

After the preliminary assessment, a sandy loam soil (sand 59%, silt 21%, clay 19%, pH 7.4, EC 1.92 dS/m) was amended with different concentrations (0.1 to 0.4%) of SHNC and the water retention at field capacity (0.03 MPa pressure) was investigated. The results shown in Figure 6 reveal that improvement in water retention capacity of the soil depends on the quantity of the SHNC used. Similar effects of polymeric gels on loamy soil water holding properties by Akhtar *et al.* [2], and in sandy soils by Bai *et al.* [23] and Dorraji *et al.* [24] have been investigated. 

The effect of SHNC on other soil quality attributes such as pH, EC, bulk density, porosity and hydraulic conductivity were also investigated for first, second and third hydration cycles and are shown in Table 1. The pH and EC varied with the application of SHNC (Table 1). The pH of the soil decreased and on the other hand EC increased with the introduction of 0.1 to 0.4% SHNC at field capacity. It seems as the reduction in the pH might have enhanced the discharge of soil inorganic salts due to which EC of the soil increased. Similar effect on the pH and EC of soils depending upon the chemical structure of the superabsorbent polymer and characteristics of the soils has been reported by Liu *et al.* [13] and Bai *et al.* [23] while studying the characteristics of chitosan-graft-poly(acrylic acid)/sodium humate superabsorbent.

Bulk density as one of the main indicators of soil quality has relationship with other properties such as porosity, moisture, and hydraulic conductivity. The maintenance of appropriate bulk density is an important objective in agriculture. Previously the decrease in soil bulk density to different degrees by the application of hydrogel was appraised by Liu *et al.* [13]. They applied chitosan-g-poly(acrylic acid)/sodium humate superabsorbent and indicated a strong relationship between moisture conditions and ameliorative effects of SHNCs on soil. The results of the present study support the earlier findings of Liu *et al*. [13]. 

The most important indicator of soil quality, *i.e.*, the hydraulic conductivity, was initially observed to be 74 mm/h (Table 1) in sandy loam soil comprising sand 59%, silt 21% and clay 19%. A remarkable decreased in hydraulic conductivity was observed with the increase in the concentration of poly(acrylamide-*co*-acrylic acid)/AlZnFe_2_O_4_ superabsorbent hydrogel nanocomposite. Similar studies were conducted by Bhardwaj *et al.* [26] in sandy soils by applying polyacrylamides. Moreover, a former study by El-Shafei *et al.* [25], regarding the influence of upper layer treatment of gel-conditioner on water movement in sandy soils under sprinkler infiltration, showed almost similar type of effects as observed in the present study. However our newly synthesized poly(acrylamide-*co*-acrylic acid)/AlZnFe_2_O_4_ superabsorbent hydrogel nanocomposite (SHNC) enhanced the moisture retention of sandy loam soils and plant available water significantly, thereby, slowing down the rate of moisture loss, due to which a delay of five to eight days in wilting point was observed. Such a delay in wilting point reduces the water requirement of plants [2,25,26,27].

## 3. Experimental

### 3.1. Materials

#### 3.1.1. Soils Used in the Experiment

Sandy loam soil (sand 58%, silt 23%, clay 19%, pH 7.5, EC 1.92 dS/m) was collected from 25 cm depth at two sites at the Postgraduate Agricultural Research Station (PARS) Jhang Road Faisalabad (31°26'N, 73°06'E), Pakistan. The soils were low in organic matter. The soils were air-dried, ground, and passed through 2 mm sieve and fractions of less than 2 mm were used in the experiments.

#### 3.1.2. Chemicals

All the chemicals including zinc sulphate, aluminum sulphate, sodium hydroxide, ferric chloride, acrylic acid acrylamide, potassium peroxydisulphate and potassium metabisulphite were analytical reagent grade purchased from Sigma Chemical Co. (St. Louis, MO, USA).

### 3.2. Methods

#### 3.2.1. Synthesis of Nano-Sized AlZnFe_2_O_4_

Nano-sized AlZnFe_2_O_4_ was prepared by the co-precipitation method. The following solutions were prepared in deionized water.

1. Al_2_(SO4)_3_·18 H_2_O          32.4 g/100 mL          Solution A
2. ZnSO_4_·H_2_O          5.4 g/100 mL          Solution B
3. FeCl_3_·6H_2_O          20.0 g/100 mL          Solution C
4. NaOH          22.0 g/200 mL          Solution D

Solutions A, B, C were mixed and stirred for one hour and heated up to 65 °C. Then solution D was added dropwise to the mixture with constant stirring. This process took two hours and reddish brown precipitates were formed. The resulting mixture was stirred continuously for half an hour and cooled to room temperature. The product thus obtained was washed and filtered repeatedly with deionized water until the chlorides were removed and the pH of the residual solution reached 7.0. The sample was then dried in an oven at 100 °C for 4 h. The dried precipitate were ground with pestle and mortar to a fine powder and calcined at 600 °C for 3 h in a muffle furnace to get nano-sized AlZnFe_2_O_4_ composite material.

#### 3.2.2. Syntheses of Poly(AA-*co*-AAm)/AlZnFe_2_O_4_ Superabsorbent Hydrogel Nanocomposite

The method of Liu and Rempel [28] with some modifications was followed for the preparation of superabsorbent hydrogel. Deionized water (450 mL) was taken in a flask fitted with a mechanical stirrer, condenser and thermometer. Acrylic acid (46 g) and acrylamide (4 g) were dissolved in the water. Triton X-100 (0.1 g) and diethylene glycol (5 g) were added to the monomer solution and stirred for 30 min.Then potassium persulfate (0.2 g) and potassium metabisulfite (0.1 g) were added into the flask and heated to 65 °C while stirring. Sodium hydroxide (1 M) solution was added to the reaction mixture to adjust the pH at 4.5. The temperature of the resulting solution was raised to 75 °C and maintained for 2 h. Then the mixture solution was cooled down to 45 °C and 37% formaldehyde (12.5 mL) was added and stirred for 30 min. Again the reaction mixture was heated to 75 °C for 2 h and cooled to room temperature. Then nano sized AlZnFe_2_O_4_ (2.5 g) was added and stirred for 15 min. The polymer thus formed was precipitated with methanol, washed with ethanol, dried at 80 °C to constant weight and ground.

#### 3.2.3. Physical Characterization

The surface morphology and size of the AlZnFe_2_O_4_ nanoparticles were studied by field emission scanning electron microscope (FE-SEM, JSM-7401F JEOL Ltd., Akishima, Japan) as shown in Figure 1. The chemical composition of the nanoparticles was determined by Energy Dispersive X-ray analysis using Inca-FET-3, Oxford Instruments (UK) Ltd (Figure 2). The SEM image of AlZnFe_2_O_4_ (Figure 3) was also taken to evaluate the morphologies of the nanoparticles. The dried SHNC samples were characterized by FTIR spectroscopy using an equipment Tensor-27, Bruker Optics (Ettlingen, Germany) in the scanning range 4,000 to 500 cm^−1^.

#### 3.2.4. Measurements of the Water Absorbency

An accurately weighed amount (0.05 g) of SHNC samples were immersed in distilled water for 4 h to reach swelling equilibrium. The swollen SHNCs were filtered out with a mesh screen. The equilibrium water absorbency of swollen samples of the SHNC was calculated with the following equation [18]:


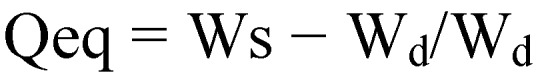


where Qeq, equilibrium water absorbency, W_d_ and Ws are weights of the dry sample and swollen sample, respectively.

#### 3.2.5. Reswelling Capability

The sample (0.05 g) was saturated in distilled water (100 mL) to attain swelling equilibrium. The swollen sample of SHNC was dehydrated at 80 °C in an oven. After complete drying, same quantity of water was added to the recovered SHNC to get the swelling equilibrium. The same method was repeated again to evaluate the reswelling capabilities of the SHNCs.

#### 3.2.6. Measurement of the Water-Retention Properties in Sandy Loam Soil

For the determination of water retention properties, SHNC was mixed uniformly with sandy loam soils to concentrations of 0, 0.1, 0.2, 0.3 and 0.4%. These SHNC-amended soils were put in ventilated paper cups and 100 mL tap water was added gradually to the cups (maintaining temperature of 25 °C and relative air humidity equal to 28%). The weights of the pots were recorded daily until no noticeable weight loss was observed. The pots were again soaked and the method was repeated for the 2nd wetting-drying cycle [2]. The water retention ratio of sandy loam soil was calculated [2,18]. 

#### 3.2.7. Measurement of Soil pH and EC

For the determination of pH and EC, soil samples were collected from 0–15, 15–30 and 30–45 cm depths (an effective root zone). Electrical conductivity (EC) and pH of soaked soil were determined by WTW conductivity meter (Model LF-530, ENVCO, Brisbane, Australia) and pH meter Corning-130 (New York, NY, USA), respectively.

#### 3.2.8. Measurement of Bulk Density and Porosity

Bulk density (the mass of a unit volume of dry soil) of soil sample was measured from all replications and from all treatments. The soil samples were collected from the depth of 0–15 cm, 15–30 cm, 30–45 cm with core auger (core sampler) and dried in oven for 24 h at 105 °C to determine its oven dry weight (Ws) and calculated soil bulk density using the following relationship: 


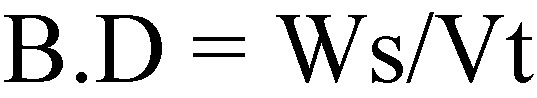


where B.D = Bulk density of soil (g/cm^3^), Ws = Weight of oven dry soil (g) and Vt = Volume of soil (cm^3^).

#### 3.2.9. Measurement of Hydraulic Conductivity

The method of Moutier *et al.*, [29] with slight modifications was followed for the measurement of hydraulic conductivity. A Perspex cylinder 10 cm long and 5 cm wide with a fine metal mesh at the bottom were used to determine the hydraulic conductivity of soil amended with 0, 0.1, 0.2, 0.3, 0.4% SHNC. The metal mesh at the bottom was covered with a 5 mm layer of coarse sand. Amended soil (150 g) was put in the cylinder, pressed to the bulk density 1.5 g/cm^3^ and covered with a filter paper. Tap water with 0.83 EC was flowed from the bottom at the rate of 40–50 mm/h with the help of a peristaltic pump. The changes in volume of soil were calculated when water level reached at the top by measuring the height of soil column. The flow direction was reversed and a hydraulic head 0.45 m was applied to leach the column with tap water. Height of soil column was monitored during leaching. The leachate was collected and hydraulic conductivity was calculated.

#### 3.2.10. Evaluation of Seed germination and Seedling Growth in Amended Soil

Four seeds of wheat (*Triticum aestivum* L.) were sown in triplicate pots filled with sandy loam soil amended with 0.1 and 0.2%, 0.3% and 0.4% w/w SHNC and placed in a growth chamber at 28 ± 2 °C. Triplicate pots of soil without SHNC were kept as a check (control). The numbers of germinated seed were noted up to two weeks to find germination. Shoot emergence was taken as an indicator of seed germination. No water was applied except the initial saturation of the pots. The experiment was finished when wilting of seedlings appeared for the first time. Shoot length and fresh weight were recorded after harvesting. The dry mass of plant material was determined after drying at 70 °C for 24 h. 

#### 3.2.11. Statistical Analysis

Data were analyzed using two-way analysis of variance ANOVA using Minitab 2000 Version 13.2 statistical software (Minitab Inc., Centre County, PA, USA) at 5% significance level.

## 4. Conclusions

Based upon the present study data, it could be concluded that novel poly(acrylamide-co-acrylic acid)/AlZnFe_2_O_4_ superabsorbent hydrogel nanocomposite had a profound effect on the physical properties of sandy loam soil. The moisture retention and porosity of the soil was remarkably increased with the application of 0.1–0.4 w/w% SHNC. The addition of SHNC significantly reduced the hydraulic conductivity and soil bulk density relative to the control at concentrations 0.1 to 0.4 w/w%. The amount of plant-available water significantly increased, leading to enhanced wheat seed germination and seedling growth in the amended soil. The soil amendment with SHNC practically ensured improvement of soil moisture retention for a longer time. These findings suggest that proper moisture level, availability of nutrients, soil type and other environmental factors may have a significant effect on the crop yield, and therefore, an appropriate moisture level should be sought through the applications of optimum concentration of SHNC to obtain maximum crop yield and productivity.
